# Hydrogenation of Furfural over Biomass-Based Electron-Deficient Co-NC Nanotube Catalyst

**DOI:** 10.3390/nano14090788

**Published:** 2024-05-01

**Authors:** Zhu Zhu, Guangyue Xu

**Affiliations:** 1 Hefei National Research Center for Physical Sciences at the Microscale, iChEM, CAS Key Laboratory of Urban Pollutant Conversion, Anhui Province Key Laboratory of Biomass Clean Energy, University of Science and Technology of China, Hefei 230026, China; 2 Institute of Energy, Hefei Comprehensive National Science Center, Hefei 230031, China

**Keywords:** furfural, cobalt, nitrogen-doped carbon, hydrogenation

## Abstract

The conversion of furfural to furfuryl alcohol is one of the most significant reactions from industrial-scale produced biomass platform molecules to value-added chemicals. In this work, biomass-based chitosan was used as both a carbon source and nitrogen source to synthesize nitrogen-doped carbon. With the addition of cobalt, the optimized 7.5Co-NC-900 catalyst had the largest surface area and the graphite nanotube structure with the least defects. It was employed for the hydrogenation of furfural to furfuryl alcohol and reached a nearly full conversion and an equivalent yield at 130 °C in 4 MPa initial H_2_. The structure–function relationship study indicated that the N could interact with the neighbor Co in this catalyst and formed an electron-deficient Co center which was in favor of the adsorption of furfural in the nanotube and had high catalytic activity. The interactions between Co and N stabilized the catalyst so that it could remain stable in five runs of catalytic reactions.

## 1. Introduction

The demand for energy has increased rapidly since the Industrial Revolution. In particular, the huge consumption of fossil resources that contributed to global environmental issues has prompted people’s search for abundant and green alternatives. Biomass, one of the world’s most abundant renewable carbon resources, could be converted into sustainable materials, chemicals, and fuels through chemical catalytic processes. Among these, plant-biomass-derived lignocellulose-based chemicals [1], such as glucose, sorbitol, ethylene glycol, and furanic compounds like furfural and 5-hydroxylmethyl furfural, could be produced over hydrolysis, hydrogenation, pyrolysis, and dehydration reactions [2,3,4,5]. These biomass-based platform chemicals were honored as building blocks for future biorefineries, and could be further transformed into a variety of value-added chemicals with potential in the future chemical, materials, and fuel industries [6].

Among these biomass platform chemicals, the production of furfural has been industrialized through the acidic hydrolysis of agricultural waste biomass containing xylose or xylan. Furfural could be further converted into various value-added chemicals such as furfuryl alcohol, tetrahydrofurfuryl alcohol, cyclopentanone/ol, and 2-methyl furan. Nearly 90% of furfural was converted to furfuryl alcohol and further converted to value-added chemicals or used as a green solvent.

In the catalytic hydrogenation of furfural to furfuryl alcohol, transition metal catalysts with a proper ability for the hydrogen activation and hydrogenation of carbonyl groups played an important role. Conventional industrial transition metal catalysts often lead to excessive hydrogenation and low selectivity [7]. Thus, the development of catalysts with the optimized support and metal for this reaction was of great significance. Zhao et al. reported ZiF-67-derived nitrogen-doped carbon nanotubes for the confining of Co nanoparticles with Co-Nx active sites as high-performance catalysts [8]. The synthesized catalyst Co-900 demonstrated excellent catalytic activity, selectivity, and stability for a wide range of biomass-derived compounds. The active site can selectively hydrogenate various biomass-derived aldehydes and ketones into value-added fine chemicals with high selectivity. Kwangjin et al. synthesized ZIF-67 into metal Co nanoparticles supported on N-doped carbon (Co@NC) by reduction [9]. The catalytic performance of furfural hydrogenation was studied. The obtained Co@NC-400-6 catalyst had the highest activity and improved the selectivity for 2-methylfuran. It can also be altered by doping Cu into ZIF-67 to produce furfuryl alcohol. With proper H_2_ treatment to minimize damage to the intrinsic surface area and pore structure, the metal organic framework can be used as a high-performance heterogeneous catalyst by maximizing the distribution of active sites. In addition, Sanchez et al. used a precious metal Ru@CNT carbon nanotube catalyst to produce 83.5% dimethylfuran at 150 °C under a hydrogen pressure of less than 20 bar [10]. This work emphasizes the importance of support in the hydrogenation of Ru and the potential applicability of the carbon nanotube as a carrier for the hydrogenation of other biological derivatives. Mironenko et al. studied the formation of active sites and the catalytic performance of Pd@C catalysts supported on carbon nanotubes (CNTs) and carbon black (CB) in 2015 [11]. The results showed that carbon support can affect the dispersion of the supported metal and its catalytic performance in the aqueous-phase hydrogenation of furfural. The hydrogenation of furfural catalyzed by a 1.5% Pd/CB catalyst at a temperature of 50 °C and hydrogen pressure of 0.5 MPa showed high selectivity (99%) for furfuryl alcohol. The 1.5% Pd@CNT catalyst was inactive at 50 °C and 0.5 MPa, but the improvement of the THFA yield at a temperature of 90 °C and pressure of 0.5 or 2.0 MPa indicated that the trend of the furan ring reduction was increased by 52%. Co and Ni are also recognized as low-cost and high-activity non-precious metals. Gong et al. used doped activated carbon-supported metal nickel (Ni@NAC) [12], which was prepared by self-assembly through a two-step calcination in nitrogen. It was used to catalyze the hydrogenation of furfural to furfuryl alcohol, and showed excellent performance after reacting for 2 h at 140 °C and 4 MPa H_2_. However, the catalytic activity of the catalyst decreased significantly after five consecutive cycles, as the conversion of furfural decreased from 99% to 33%. Recently, Thongratkaew et al. studied the solvent effect of furfural hydrogenation under the action of a CuAl_2_O_4_ catalyst, and found that the automatic stratification of the furfural alcohol product can be achieved by using an alkane solvent, and the final yield was as high as 96% [13]. Hou et al. developed a series of Ni- and Ni_2_P-based catalysts for furfural hydrogenation. They found that the selectivity of furfural can be effectively controlled by using a Ni_2_P catalyst. Using Ni_2_P/MgO-Al_2_O_3_ as a catalyst, 95% furfural conversion and 98.3% furfural selectivity can be achieved at 398 K. Studies have shown that surface acidity affects the adsorption of furan rings and aldehyde groups on the catalyst surface [14].

In recent years, the heteroatom (B, N, O, P, etc.)-doped carbon materials, especially nitrogen-doped heterocarbon materials, have attracted much attention. The special conductivity, d-band density, and adjustment of the Fermi level of the supported metal core made it become a new catalyst carrier used in various kinds of reactions, such as hydrogenation, oxidation, and photocatalysis [15,16]. The doping of nitrogen into a carbon framework was a facile process due to the similar covalent radius of carbon (0.77 Å) and nitrogen (0.74 Å) [17]. Meanwhile, the higher electronegativity of the extra nitrogen (3.04 vs. 2.55 for carbon) would lead to the variation of the electronic structures of carbon and, therefore, enhanced the surface acidity/alkalinity, electron transfer ability, and catalytic activity [18]. Furthermore, the nitrogen content, surface area, pore size, and volume, as well as the morphologies of nitrogen-doped carbon materials, could be easily altered, so as to give them adjustable properties to meet wide applications [19].

At present, the main raw materials for the synthesis of nitrogen-doped carbon were still non-renewable fossil materials, such as melamine and ethylenediamine [20]. Biomass, renewable and abundant, could be an excellent substitute for the synthesis of nitrogen-doped carbon. Various carbon materials, such as porous carbon, 1-layer porous carbon, carbon quantum dots, heteroatom-doped porous carbon. and carbon fiber [21,22,23], have been reported to be synthesized from lignocellulosic-biomass-based agricultural wastes like rice husk, bagasse, wheat straw, and cotton straw. The nitrogen-containing biomass, such as shrimp shells, algae, bean dregs, and chitosan, could also be employed to synthesize nitrogen-doped carbon [24]. In addition, the carbon materials prepared from biomass with a unique three-dimensional structure would also have special morphologies and structures, which can promote mass transfer and improve adsorption [25,26]. For example, Gao et al. used chitin as the raw material for pyrolysis in an inert atmosphere at 800 °C, and found that the rate of pyrolysis is the key to determining the two-dimensional fold structure and nitrogen-doping type of the product. They obtained two-dimensional folded carbon nanosheets with multiple pores and nitrogen-enriched species under optimized conditions. The oxygen reduction reaction (ORR) shows the high activity, selectivity, and stability of more than 10,000 cycles [27].

During the synthesis of nitrogen-doped carbon, the addition of a transition metal could allow the attachment of the transition metal and nitrogen. This interaction could build metal sites co-ordinated by nitrogen and show special catalytic performance. Chitosan was a widely existing nitrogen-containing biomass-derived feedstock. The physicochemical properties and polymerization degree of chitosan varied significantly with the different sources, and its price also changes. In this work, a series of carbon-nitride-based Co catalyst materials were synthesized using chitosan, a nitrogen-containing biomass-derived feedstock. This Co-NC catalyst was employed in the hydrogenation of furfural to furfuryl alcohol. The structure–function relationship was studied by both theoretical calculations and experimental characterizations to find the electron-rich Co conjugated with pyrrole N was the key catalytic site for the hydrogenation reaction.

## 2. Materials and Methods

### 2.1. Materials

All chemicals were purchased from Sinopharm Chemical Reagent Co., Ltd., Shanghai, China. Chitosan (catalog number: 69047438) was biological reagent; others were analytical reagent. Pure water (conductivity less than 2 μs/cm) was purchased from Hangzhou Wahaha Group Co., Ltd., Hangzhou, China. Gases (99.999%) were purchased from Nanjing Special Gas Factory Co., Ltd., Nanjing, China. All chemicals were used directly without any further treatment.

### 2.2. Catalyst Preparation

The Co-NC catalysts were synthesized by the following procedure. Chitosan and CoCl_2_·6H_2_O with designed mass ratio were mixed in an agate mortar and were ground for 30 min. Then, the pink powder was placed into a tubular furnace (TFH-1200, Anhui CHEM^N^ Instruments Co., Ltd., Hefei, China.) to be calcined in 100 sccm NH_3_ flow. The process was shown in Figure S1.

### 2.3. Catalyst Characterization and Calculation Method

X-ray powder diffraction (XRD) was conducted on a Rigaku Smartlab X-ray diffractometer (Tokyo, Japan) with the Cu-Ka radiation. Morphologies and structures of the powders were observed by scanning electron microscopy (SEM, Zeiss GeminiSEM 450, Oberkochen, German) and transmission electron microscopy (TEM, JEOL JEM-2011, Tokyo, Japan). X-ray photoelectron spectrometer (Thermo Fisher escalab 250xi, Waltham, MA, US), Fourier-transform infrared spectrometer (FT-IR, Thermo Fisher Nicolet 8700, Waltham, MA, USA), and laser Raman spectrometer system (Horiba LabRAM HR, Kyoto, Japan) were used to study the chemical properties. The Brunauer–Emmett–Teller (BET) specific surface area was estimated by using the adsorption data obtained in Tristar II 3020 M, Mircomeritics, Atlanta, GA, USA. The temperature-programmed desorption of H_2_ (H_2_-TPD) was performed on an automatic chemical adsorption instrument (VDsorb 91i, Quzhou Vodo Instrument Co., Ltd., Quzhou, China).

The dispersity was calculated as:

$$d=\frac{molar\;amount\;of\;surface\;Co\;by\;TPD}{molar\;amout\;of\;total\;Co\;by\;ICP}$$

The TOF based on total Co was calculated as:

$$TOF=\frac{n_{production\;of\;furfuryl\;alcohol}}{n_{Total\;Co}\times Reaction\;time}$$

The TOF* based on surface Co was calculated as:

$${TOF}^{*}=\frac{n_{production\;of\;furfuryl\;alcohol}}{n_{Surface\;Co}\times Reaction\;time}$$

All TOF values were calculated using the data with conversion below 30% at 100 °C.

### 2.4. Computation Methods

All the calculations were carried out by using Materials Studio program by BIOVIA Corp. Dmol^3^ code [28,29] was used to optimize the structure of catalysts with DND 3.5 basis set and the GGA PBE functional. Co (111) facet with (4 × 4 × 1) super cell, and CoO (111) facet with (4 × 4 × 1) super cell were chosen as model for Co and Co_x_O_y_, respectively. Three layers of graphene with first layer doped by N and Co were chosen as model for planar graphite Co-NC. The (12, 0) carbon nanotube doped by N and Co were chosen as model for nanotube Co-NC. N was pyrrole-form-bonded with a Co-O atom set. All the terminal atoms were saturated by H atom, which were not shown in figures in order to be clear. Twelve of empty bands were used when calculating the density of state. Adsorption energy was calculated using the following equation:

E_adsorption_ = E_total_ − E_catalyst_ − E_surface_

### 2.5. Reaction Test

In a typical reaction, furfural and catalyst were put into a 25 mL NSC-type autoclave (Anhui CHEMN Instruments Co., Ltd.) with 10 mL of pure water as solvent. After being purged for 3 times with H_2_, the reactor was charged with H_2_ to the set pressure. Then, the reactor was heated up to reaction temperature with 10 °C/min ramping rate and kept for a certain time. After reaction, the reactor was cooled down to room temperature and n-hexanol was added as internal standard. The liquid product was analyzed by Shimadzu Nexis 2030 (Kyoto, Japan) gas chromatography (GC). The solid residue was washed with pure water for the next-run usage.

## 3. Results

### 3.1. Catalyst Characterizion

**Figure 1:**
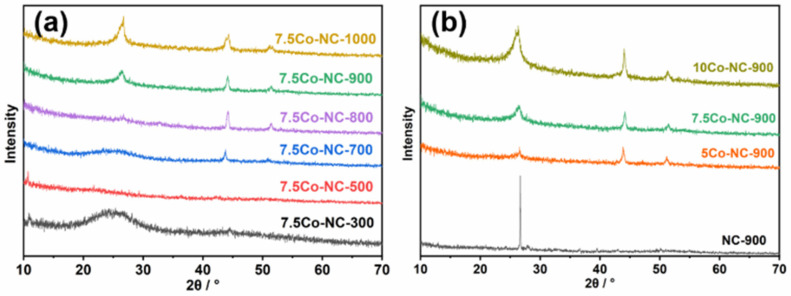
XRD patterns of Co-NC catalysts synthesized with various calcination temperatures (**a**) and Co loadings (**b**).

The xCo-CN-y catalysts were synthesized by the co-pyrolysis method with x% Co content and calcined at y °C; see details in Experimental Section. The prepared catalysts were characterized to study its physical and chemical properties. In XRD patterns, as shown in Figure 1a, the Co-NC calcined at 300 °C showed a broad diffraction peak at approximately 25°, indicating amorphous carbon. When the calcination temperature increased to 700 °C, the diffraction peak at 26° was attributed to graphite carbon. The higher the calcination temperature is, the sharper the peak became. Previous works in literature also proved that the partial graphitization of chitosan began at 700 °C. The diffraction peak of cubic cobalt, 44° for the (111) facet and 52° for the (200) facet, appeared under 700 °C and a higher calcination temperature [30]. It indicated that a higher calcination temperature would lead to the growth of Co nanoparticles. Figure 1b showed XRD patterns with different Co loadings. The NC-900 without a Co addition showed a very narrow and high graphite signal at 26°. After Co loading, the graphite peak became lower and wider, indicating that the Co addition affected the crystallization of graphite carbon and, thus, leading to a small crystal size.

**Figure 2:**
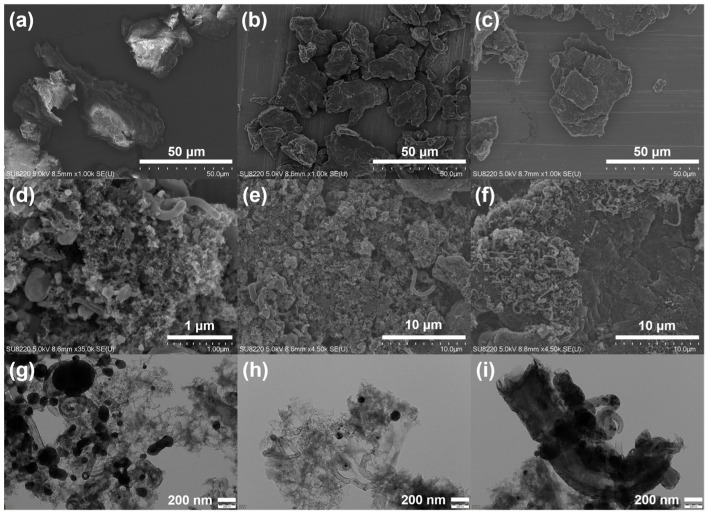
SEM images of 7.5Co-NC catalysts calcined at (**a**) 300 °C, (**b**) 500 °C, (**c**) 700 °C, (**d**) 800 °C, (**e**) 900 °C, and (**f**) 1000 °C; and TEM images of 7.5Co-NC catalysts calcined at (**g**) 800 °C, (**h**) 900 °C, and (**i**) 1000 °C.

The morphologies of the catalysts were then studied by SEM and TEM, as shown in Figure 2. The Co-NC calcined at low temperature (300–700 °C, Figure 2a–c) showed block-like structures with irregularly stacked multi-layer sheet morphologies. The particle size increased with the increase in calcination temperature. When the calcination temperature increased to 800 °C, the catalyst revealed some spongy morphology features and tube-like structures (Figure 2d). With a further increasing in calcination temperature, the pore structure of the catalyst became more compact (Figure 2e,f) and some of crystal was broken into pieces. The TEM images could also show the impact of calcination temperatures. The carbon nanotube grew with the increase in the calcination temperature (Figure 2g–i). Moreover, the wall of carbon nanotube was thicker in the catalyst calcined at 1000 °C. Some large-sized Co nanoparticles could be observed in the catalyst calcined at 800 °C and 1000 °C, while only few small-sized Co nanoparticles were shown in the catalyst calcined at 900 °C.

**Figure 3:**
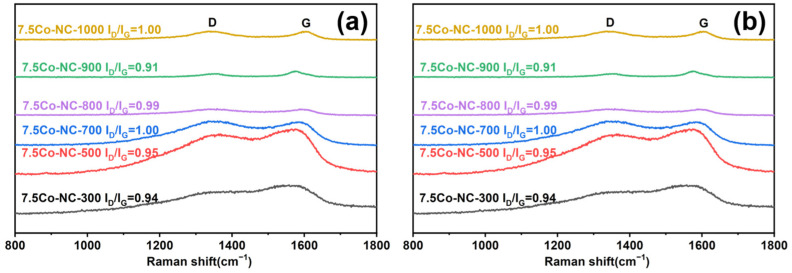
Raman spectra of Co-NC catalysts synthesized with various calcination temperatures (**a**) and Co loadings (**b**).

Figure 3 showed the Raman spectra of various Co-NC catalysts. All samples showed a D peak at 1350 cm^−1^ and a G peak at 1595 cm^−1^. The D peak showed the defects and disordered carbon, while the G peak showed the ordered graphite sp^2^ C=C bond [31]. The ratio of peak intensity of the D peak and G peak (I_D_/I_G_) indicated the structural defects of carbon materials, and was labelled in the Figure 3 [32]. A smaller I_D_/I_G_ parameter demonstrated a higher degree of graphitization with less defects in the carbon. The I_D_/I_G_ showed an inverted volcano-like variation trend within a 700–1000 °C calcination temperature and reached the lowest at 900 °C. It indicated a well-grown carbon structure at 900 °C, which is consistent with the electron microscope observations. The I_D_/I_G_ ratio decreased with the increase in Co content. It indicated that the addition of Co could improve the formation of graphite carbon and prevent the defects.

### 3.2. Catalytic Reactions

**Figure 4:**
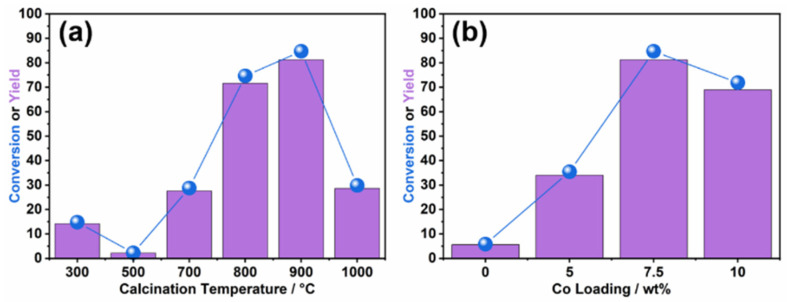
Catalytic conversions of furfural and yield of furfuryl alcohol over different Co-NC catalysts synthesized with various calcination temperatures (**a**) and Co loadings (**b**). Reaction conditions: 40 mg of furfural, 10 mg of catalysts, 10 mL of water, and 100 °C in 4 MPa initial H_2_ for 4 h.

Then, the catalysts were tested in the hydrogenation of furfural, as shown in Figure 4, Table 1 and Table 2. The calcination temperature had a great influence on the conversion of furfural to furfuryl alcohol. At a calcination temperature as low as 300 °C, the conversion over 7.5Co-NC-300 was 15%. When the calcination temperature increased to 500 °C, the conversion and yield over 7.5Co-NC-500 decreased to 2%. A further increase in the calcination temperature would lead to a remarkable promotion in catalytic hydrogenation. It could reach an 81% yield of furfuryl alcohol over 7.5Co-NC-900. However, 7.5Co-NC-1000 could only obtain a 29% yield. These results matched well with the characterizations above. The 7.5Co-NC-900 had the best grown nitrogen-doped carbon structure with nanotube morphologies and the Co dispersed well in this catalyst. The content of Co loading also had an impact on the catalytic performance. The 7.5Co-NC-900 showed a better conversion and yield than 5Co-NC-900 and 10Co-NC-900. The proper content of metal could not only maximize the dispersion of metal, but also affected the structure of nitrogen-doped carbon. The catalyst was evaluated by TOF based on total Co (apparent TOF) and TOF* based on surface Co (intrinsic TOF*). The latter revealed the intrinsic activity of Co. It can be seen that the 7.5Co-NC-900 revealed the highest TOF and TOF*. The reaction conditions were optimized over the 7.5Co-NC-900 catalyst. A higher reaction temperature and initial hydrogen pressure would both enhance the furfural conversion and furfuryl alcohol yield. The best result was a >99% conversion with a >99% yield at 130 °C and 4 MPa for 2 h. During all these reactions, the conversion of furfural and the yield of furfuryl alcohol were all similar, indicating that there were barely any side reactions.

**Figure 5:**
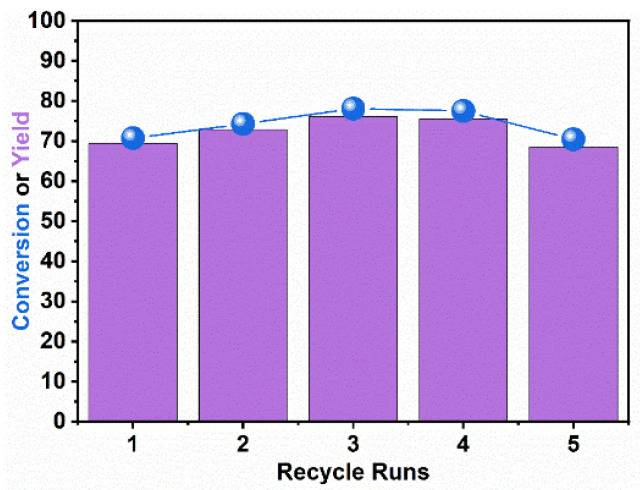
Recycling test. Reaction conditions: 40 mg of furfural, 10 mg of 7.5Co-NC-900, and 10 mL of water 120 °C in 4 MPa initial H_2_ for 1 h.

The reusability of the 7.5Co-NC-900 catalyst was tested in an incomplete conversion stage at 120 °C in 4 MPa initial H_2_ for 1 h. During the five recycle runs, the conversion of furfural and the yield of furfuryl alcohol both remained steady. It indicated that the Co sites and nitrogen-doped carbon were very stable in the catalytic reactions (Figure 5).

### 3.3. Structure–Function Relationship

**Figure 6:**
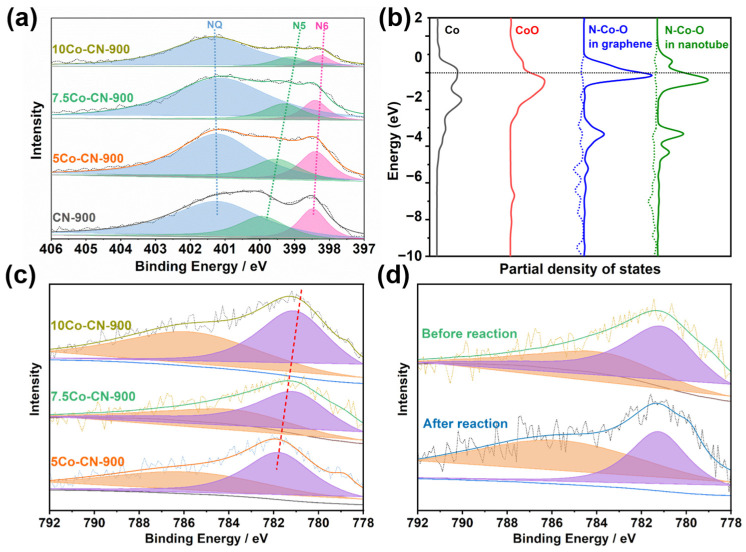
(**a**) Deconvoluted XPS spectra of N1s in Co-NC catalysts. (**b**) Partial density of states (pDoS) in various models. The solid line in pDoS was Co 3d while the dashed line was N 2p. Fermi energy was set as 0 eV. (**c**) Deconvoluted XPS spectra of Co 2p in Co-NC catalysts. (**d**) Deconvoluted XPS spectra of Co 2p in fresh and used 7.5Co-NC-900 catalysts.

To further study the structure–function relationship, XPS, and adsorbed FT-IR, as well as DFT calculations were employed. The XPS spectra of N 1s was deconvoluted to three predominate distinct peaks, tetraco-ordinated N (NQ), pyrrole-like N in five-membered rings (N5), and pyridine-like N in six-membered rings (N6). The original N in chitosan were all tetraco-ordinated amino N. Therefore, there were large amounts of the tetraco-ordinated N residual in catalysts. The amino N, as well as the NH_3_ atmosphere gas during calcination, would both be doped to the carbon skeleton via pyrolysis processes and formed pyrrole-like N in five-membered rings and pyridine-like N in six-membered rings. Figure 6a indicated that the binding energy of N5 decreased with the increase in Co loading, while that of NQ and N6 almost remained unchanged. It certified that the Co interacted with the pyrrole-like N in this catalyst. In this strong interaction, the N atom attracted electrons from the neighbor Co atoms and made N-Co and N-Co-O sites with the electron–deficient Co center. The state of the outer electrons (third electron) in Co atoms were between that of Co and CoO. The DFT calculations of the partial density of states in four models also proved the phenomenon. As some electrons were donated to neighbor N atoms, more free unoccupied d orbitals were released and the d-band center was closer to the Fermi level. The higher d-band center offered higher catalytic activities. Meanwhile, the overlap of N 2p and Co 3d in the bonding orbital region indicated the formation of a molecular orbital of the N-Co bond, which agreed with the XPS results that the Co and N had very strong interactions. It also agreed with the results that the state of the electron of the Co element was between Co and CoO, and the binding energy of Co 2p decreased gradually with the increase in Co content. This interaction not only stabilized the catalysts from leaching in the catalytic reactions, but also increased the catalytic activities of the Co center. There was no significant change in the XPS spectra of Co before and after the reaction. It proved that Co remained stable during the reaction, which was consistent with the results of the recycling test.

**Figure 7:**
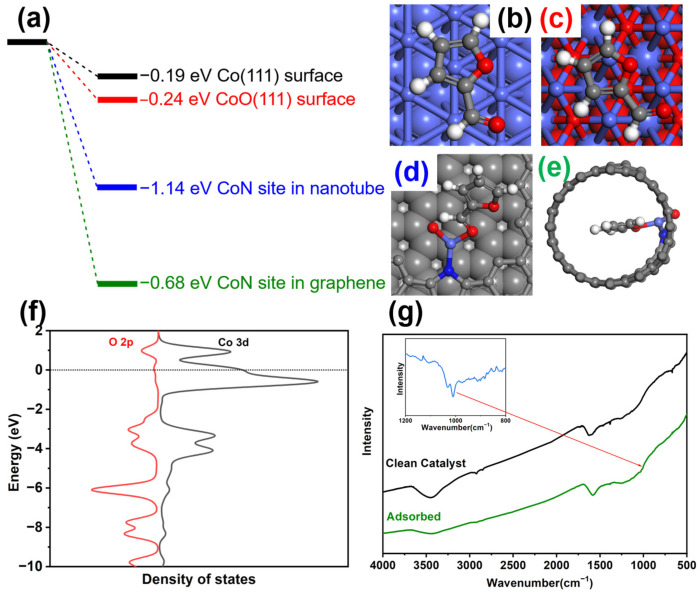
(**a**) Adsorption energy of furfural in various models. (**b**–**e**) Adsorption models. Co: light blue; O: red; N: dark blue; C: grey; H: white. (**f**) pDoS of Co 3d in catalyst and O 2p in the aldehyde group of furfural. (**g**) FT-IR spectra of clean 7.5Co-CN-900 before and after being adsorbed with furfural; inner: difference between two spectra.

The adsorption of feedstock was always one of the key factors that affected the catalytic activity in heterogeneous catalytic reactions. From the DFT calculation results, furfural was energetically more favorable to be adsorbed on N-Co-O sites, as shown in Figure 7a–e. The nanotube could adsorb furfural inside the channel and reach a more stable state. The 7.5Co-NC-900 catalysts had nanotube-like morphologies, and, therefore, easily capture the furfural molecule in the tube. The DFT calculations of the partial density of states in this adsorption model in Figure 7f also demonstrated the interactions between Co in the catalyst and O in the aldehyde group of furfural. The large overlap in the bonding orbital region exhibited the formation of a Co-O bond in the chemical adsorption. The adsorbed FT-IR experiment also proved the adsorption of furfural in the catalyst, as shown in Figure 7g. After washing out all the weak adsorbed or physical adsorbed furfural, there was still a different new peak at 1020 cm^−1^, indicating the C-H peak of C_3_ and C_4_ in the furan rings. Other peaks in the original catalyst all remained unchanged, such as 1376 cm^−1^ for the stretching peak of C-N in the tetraco-ordinated amino groups, 1470 cm^−1^ for the C=C vibration peak of the benzene ring in carbon, and 1590 cm^−1^ and 1620 cm^−1^ for the C=C/C=N stretching vibration peak of the graphite carbon or nitrogen-doped carbon, respectively. Based on the above experimental characterizations and theoretical calculations, the N could interact with the neighbor Co in this catalyst and form an electron–deficient Co center. This Co center was in favor of the adsorption of furfural in the nanotube and had high catalytic activity. Moreover, the interactions between Co and N stabilized the catalyst so that it could remain stable in catalytic reactions.

## 4. Conclusions

In this work, biomass-based chitosan was used as both a carbon source and nitrogen source to synthesize nitrogen-doped carbon. The 7.5Co-NC-900 catalyst with 7.5% Co loading and calcined at 900 °C had the largest surface area and graphite nanotube structure with the least defects. The hydrogenation of furfural to furfuryl alcohol reached a >99% conversion and yield at 130 °C in 4 MPa initial H_2_ over the 7.5Co-NC-900 catalyst. The structure–function relationship study indicated that the N could interact with the neighbor Co in this catalyst and form an electron–deficient Co center which was in favor of the adsorption of furfural in the nanotube and had high catalytic activity. The interactions between Co and N stabilized the catalyst so that it could remain stable in five runs of the catalytic reaction. This catalytic system realized a typical biomass conversion reaction over the biomass-derived catalyst. The catalyst was easy to prepare on a large scale. It was stable and non-expensive. It could have potential for industrial applications.

## Figures and Tables

**Table 1:** Dispersity of Co in various catalysts, the TOF based on total Co, and the TOF* based on surface Co ^a^.

Entry	Catalyst	Dispersity/%	TOF/h^−1^	TOF*/h^−1^
1	7.5Co-NC-300	4.1	1.1	27.8
2	7.5Co-NC-500	4.9	0.1	3.6
3	7.5Co-NC-700	7.4	2.2	30.0
4	7.5Co-NC-800	8.7	8.7	99.1
5	7.5Co-NC-900	7.1	12.1	170.7
6	7.5Co-NC-1000	1.6	2.3	144.7
7	5Co-NC-900	7.3	4.1	56.0
8	10Co-NC-900	4.8	6.1	126.4

^a^ The calculation method was described in the Experimental Section. All TOF data were calculated based on reaction conditions of 40 mg of furfural, 10 mg of catalysts, 10 mL of water, and 100 °C in 4 MPa initial H_2_, and the conversions were controlled to be below 30%.

**Table 2:** Optimization of catalytic reaction conditions ^a^.

Entry	T/°C	P/MPa	T/h	Conv./%	Yield/%	Carbon Balance/%
1	110	4	1	43	41	98
2	120	4	1	73	70	97
3	130	4	1	91	87	97
4	120	0	1	0	0	100
5	120	1	1	29	29	100
6	120	2	1	35	34	99
7	120	3	1	51	50	99
8	120	4	1	73	70	97
9	120	4	0	0	0	100
10	120	4	0.5	39	38	99
11	120	4	1	73	70	97
12	120	4	2	85	82	97
13	130	4	2	>99	>99	100

^a^ 40 mg of furfural, 10 mg of 7.5Co-NC-900 catalyst, and 10 mL of water.

## Data Availability

Data are contained within the article and supplementary materials.

## Supplementary Materials

The following supporting information can be downloaded at: https://www.mdpi.com/article/10.3390/nano14090788/s1, Figure S1: Synthesis procedure of Co-NC catalysts; Figure S2: XRD patterns of Co-NC catalysts synthesized with various calcination temperatures and Co loadings; Figure S3: SEM patterns of Co-NC catalysts synthesized with various Co loadings at 900 °C; Figure S4: Raman spectra of Co-NC catalysts synthesized with various calcination temperatures and Co loadings; Figure S5: Infrared spectra of Co-NC catalysts synthesized with various calcination temperatures and Co loadings; Table S1: The content of nitrogen in various catalysts.
