# Supplementary material for: Hydrogenation of Furfural over Biomass-Based Electron-Deficient Co-NC Nanotube Catalyst
Source: Nanomaterials (Basel). 2024 May 1;14(9):788. doi: 10.3390/nano14090788 (PMC11085387; doi:10.3390/nano14090788)
Supplement: Supplementary file 1 [file nanomaterials-14-00788-s001.zip › nanomaterials-2983603-supplementary.pdf]

Supporting Information

Hydrogenation of furfural over biomass-based electron-deficient Co-NC nanotube catalyst

Zhu Zhu[1], Guangyue Xu*[1]

1. Hefei National Research Center for Physical Sciences at the Microscale, iChEM, CAS Key Laboratory of Urban Pollutant Conversion, Anhui Province Key Laboratory of Biomass Clean Energy, University of Science and Technology of China, Hefei 230026, China. Institute of Energy, Hefei Comprehensive National Science Center, Hefei 230031.

*Email:

GY Xu: craneyue@mail.ustc.edu.cn

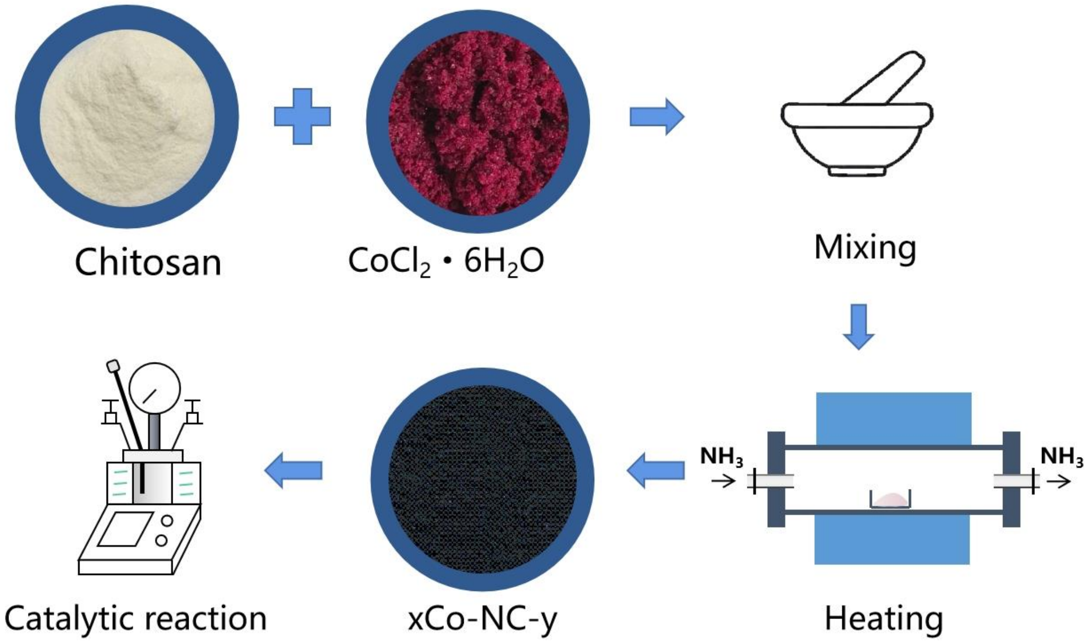

Figure S1. Synthesis procedure of Co-NC catalysts.

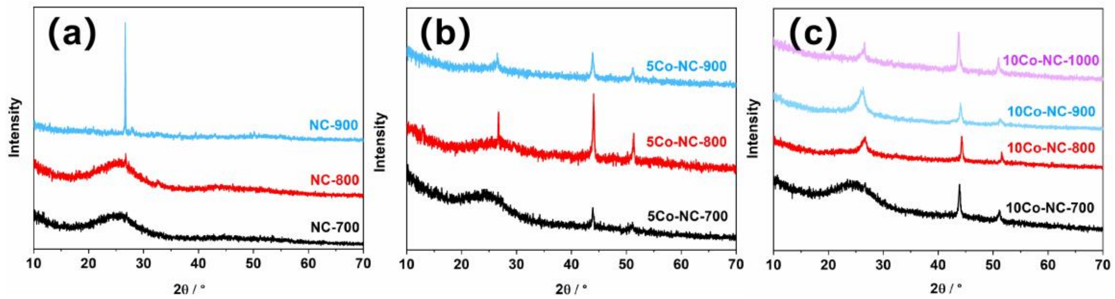

Figure S2. XRD patterns of Co-NC catalysts synthesized with various calcination temperatures and Co loadings.

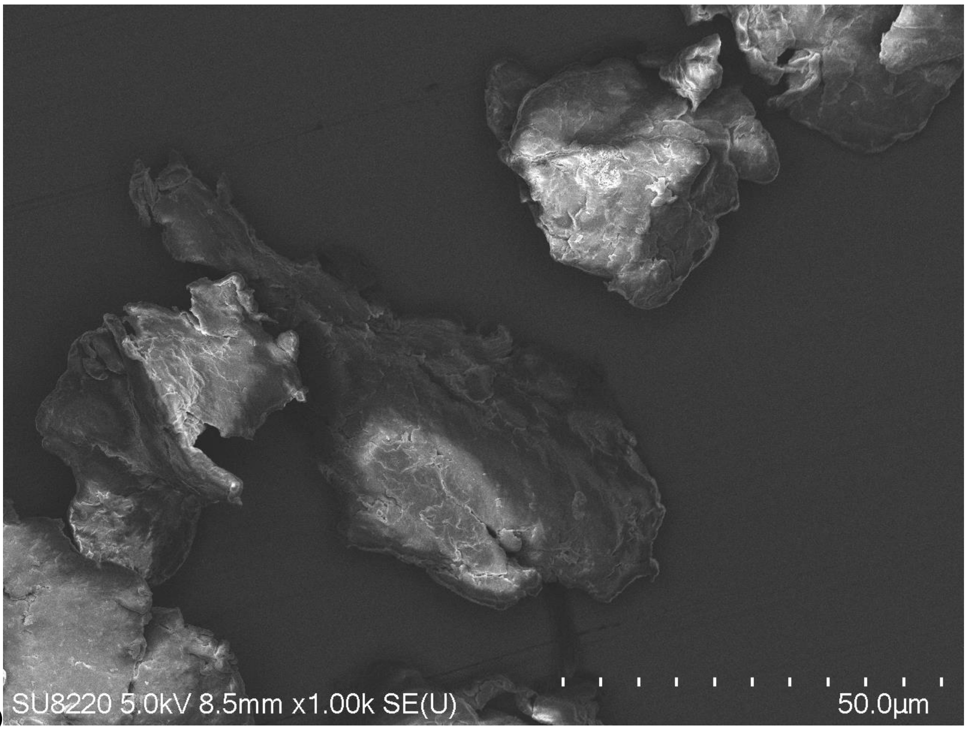
SU8220 5.0kV 8.5mm x1.00k SE(U)
50.0μm

(a)

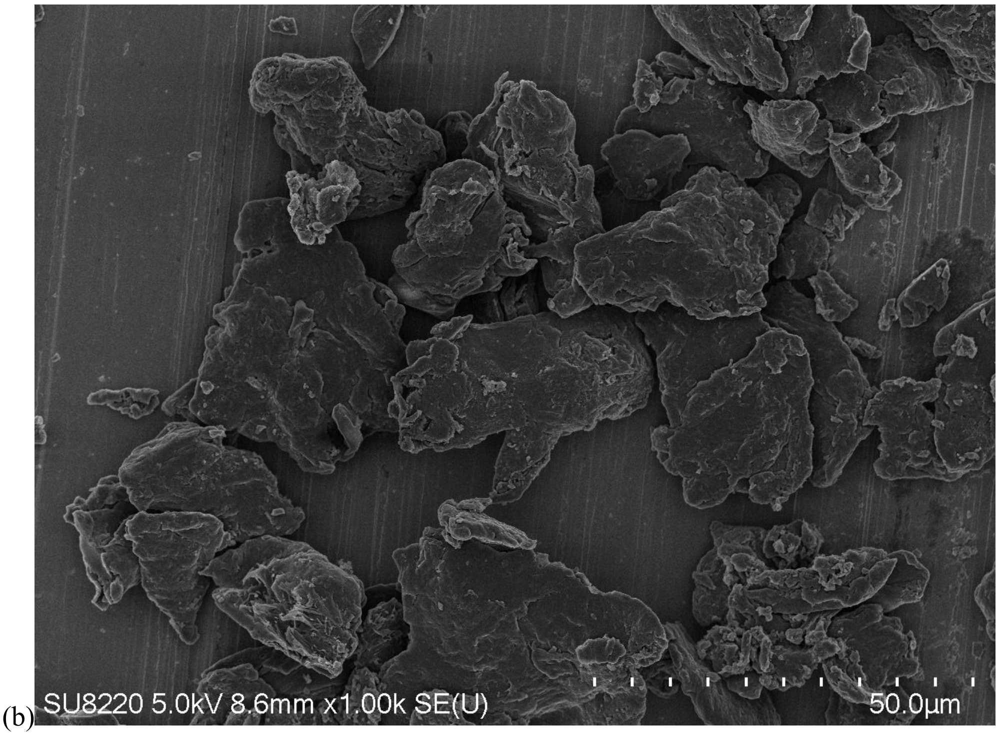
SU8220 5.0kV 8.6mm x1.00k SE(U)
50.0μm

(b)

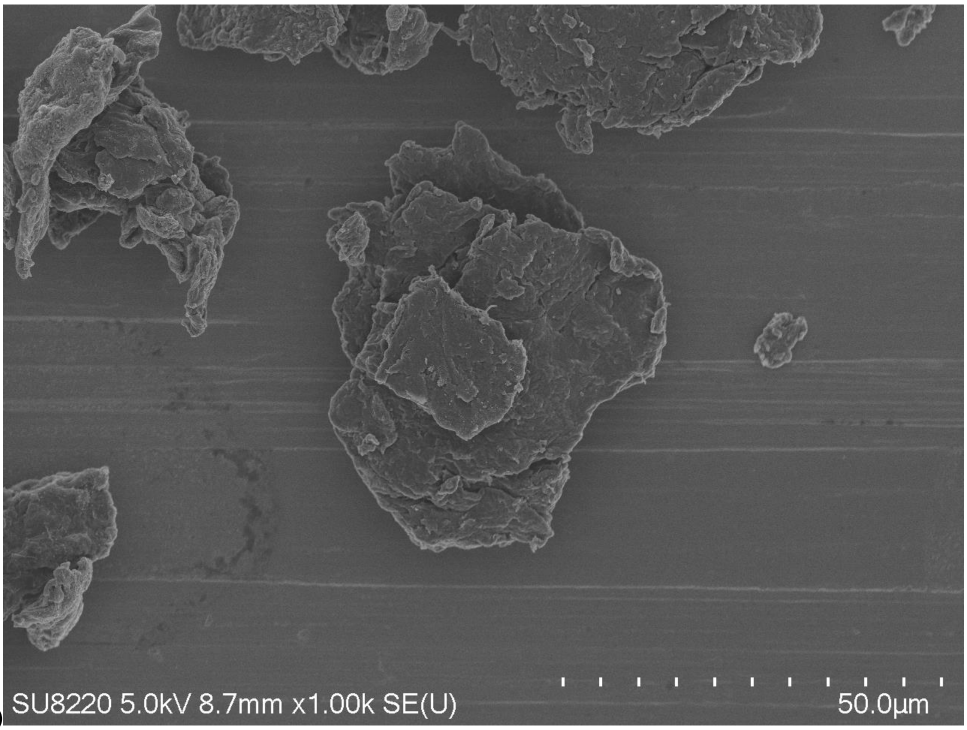
SU8220 5.0kV 8.7mm x1.00k SE(U)
50.0μm
(c)

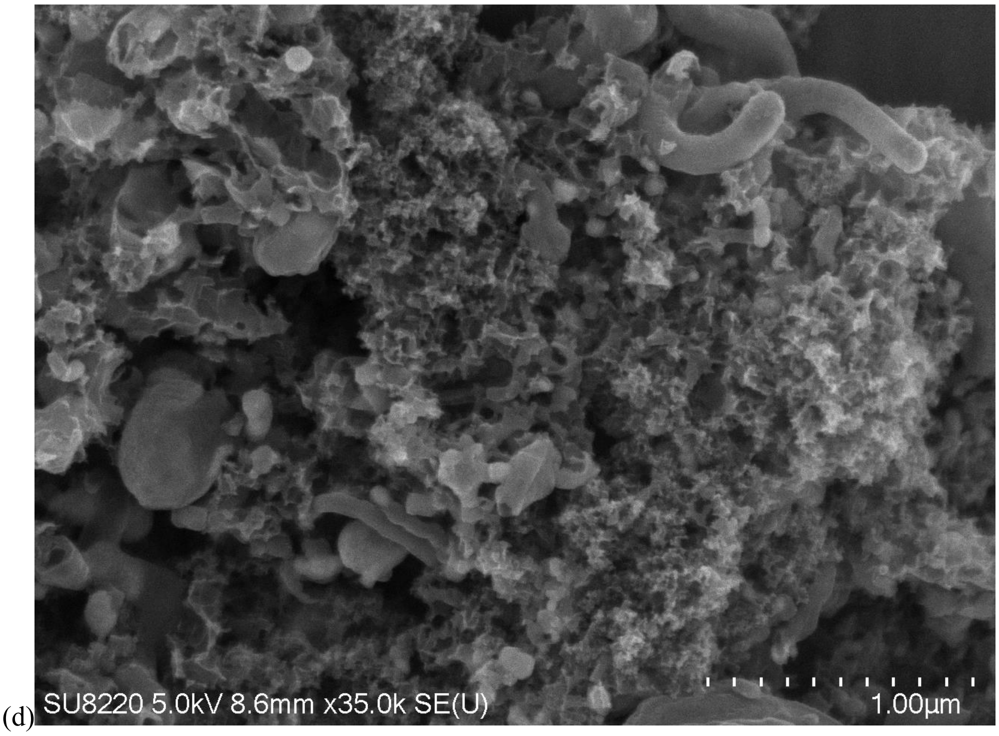
SU8220 5.0kV 8.6mm x35.0k SE(U)
1.00μm
(d)

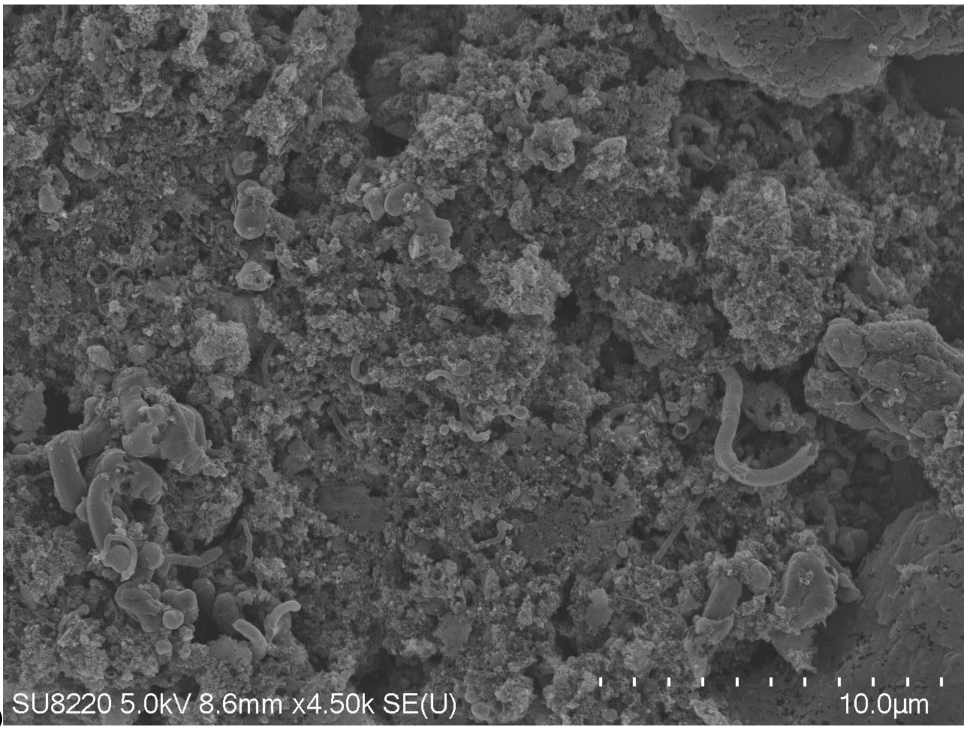
SU8220 5.0kV 8.6mm x4.50k SE(U)
10.0µm
(e)

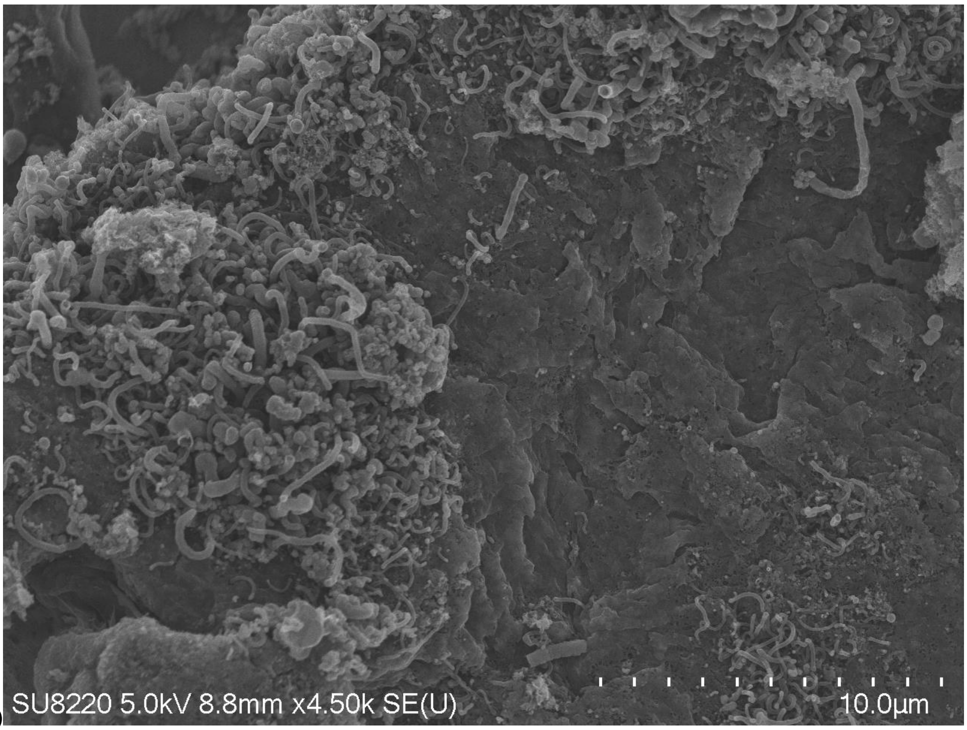
SU8220 5.0kV 8.8mm x4.50k SE(U)
10.0µm
(f)

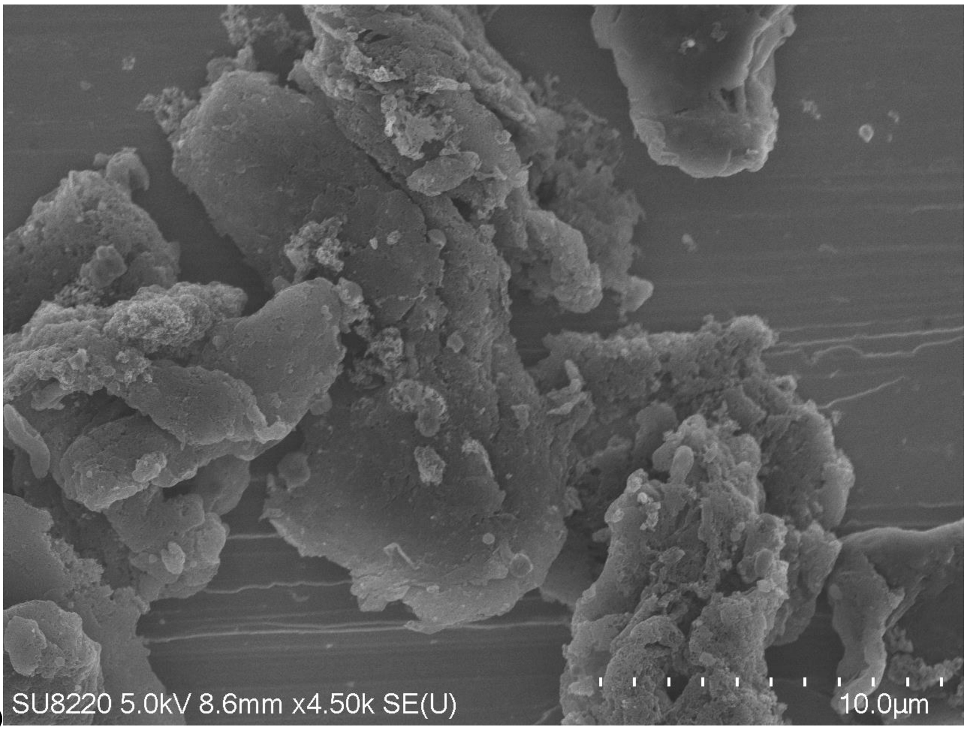
SU8220 5.0kV 8.6mm x4.50k SE(U)
10.0μm
(g)

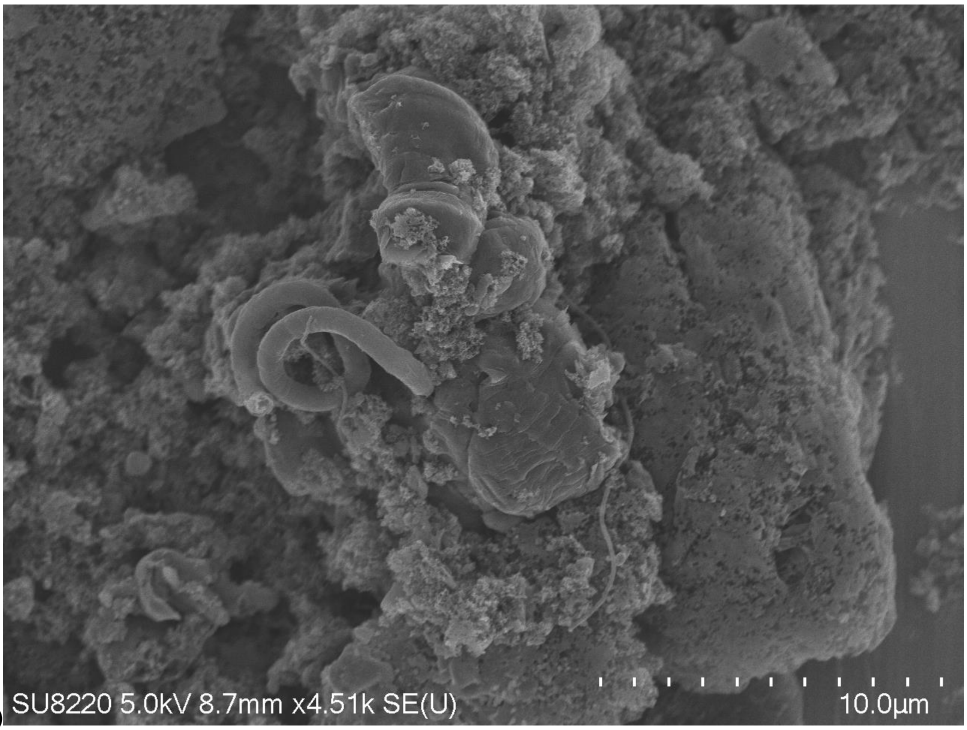
SU8220 5.0kV 8.7mm x4.51k SE(U)
10.0μm
(h)

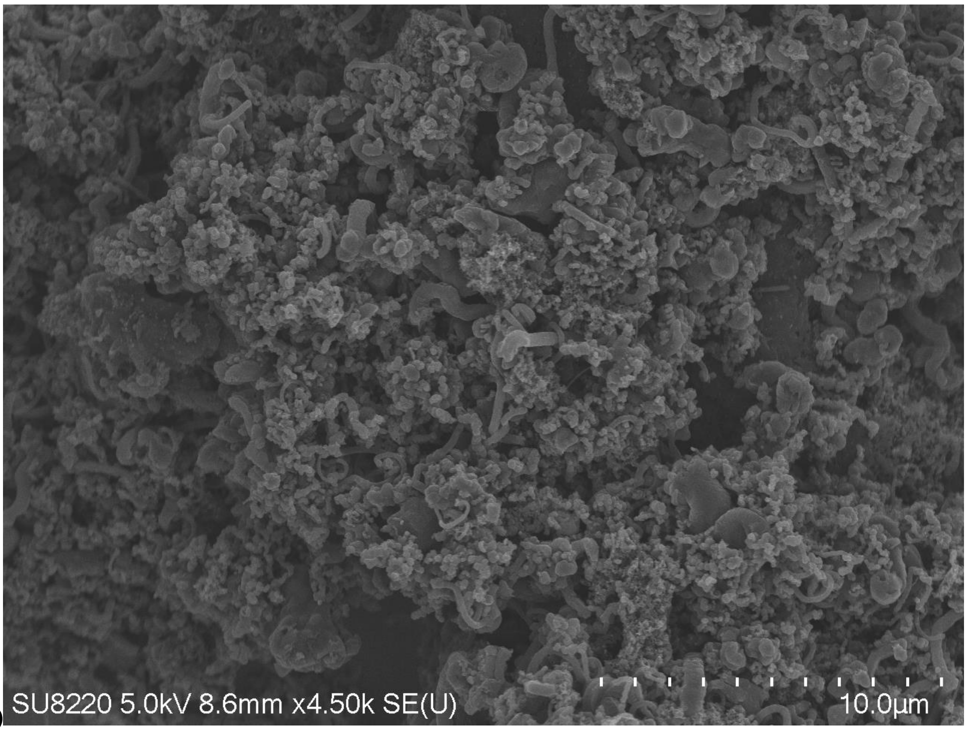

(i)

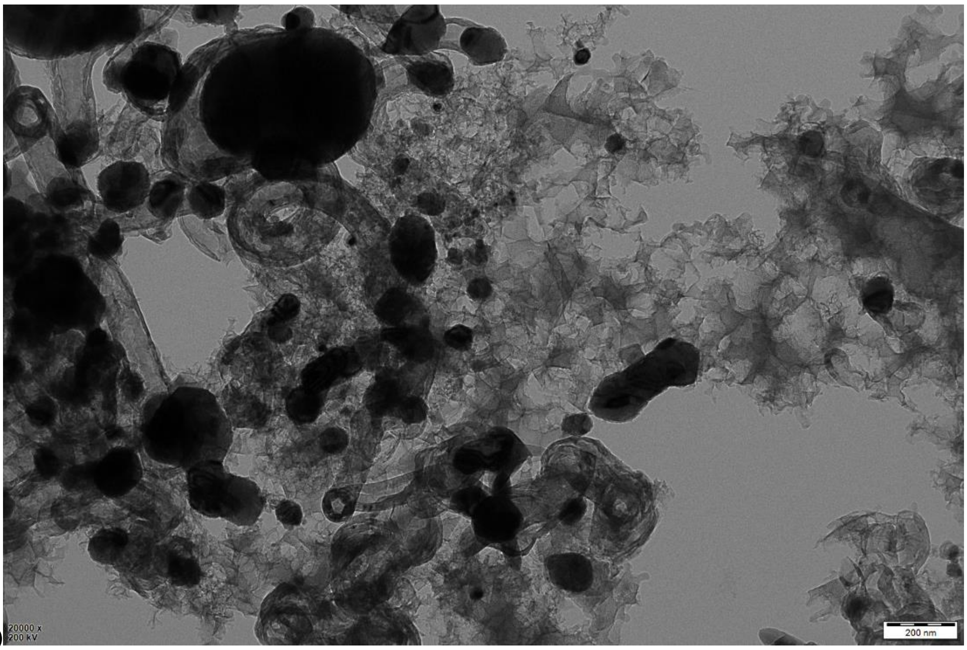

(j)

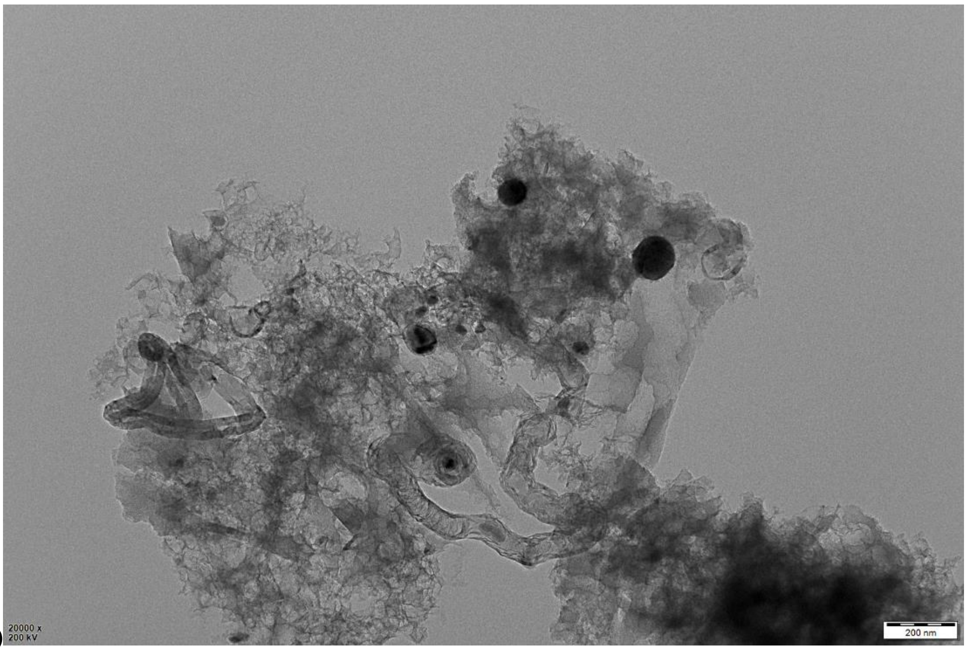

(k)

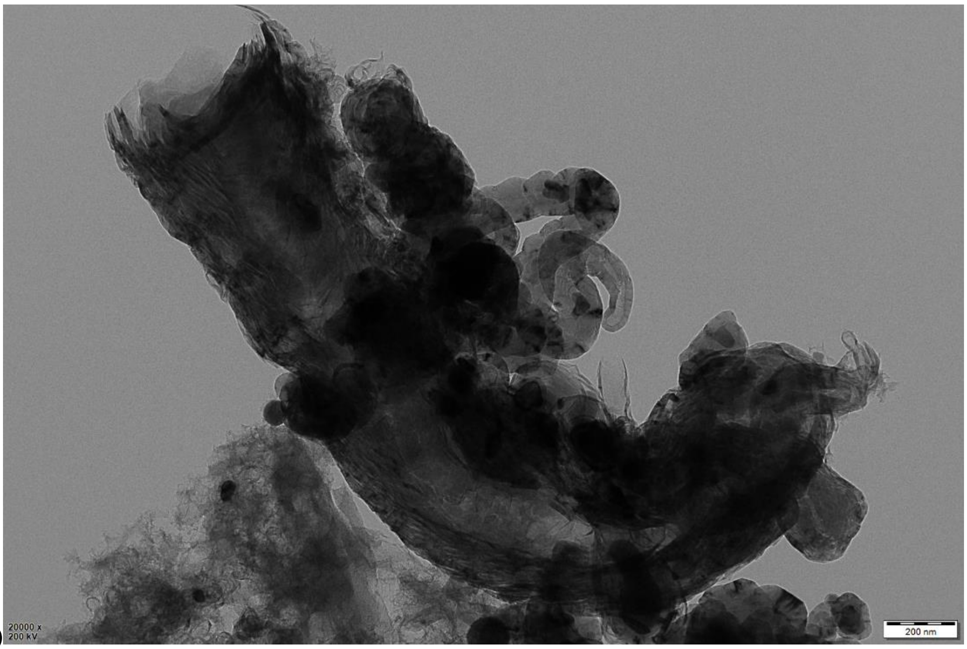

(l)

Figure S3. SEM images of (a) 7.5Co-NC-300, (b) 7.5Co-NC-500, (c) 7.5Co-NC-700, (d) 7.5Co-NC-800, (e) 7.5Co-NC-900, (f) 7.5Co-NC-1000, (g) 0Co-NC-900, (h) 5Co-NC-900, (i) 10Co-NC-900; and TEM images of (j) 7.5Co-NC-800, (k) 7.5Co-NC-900, (l) 7.5Co-NC-1000.

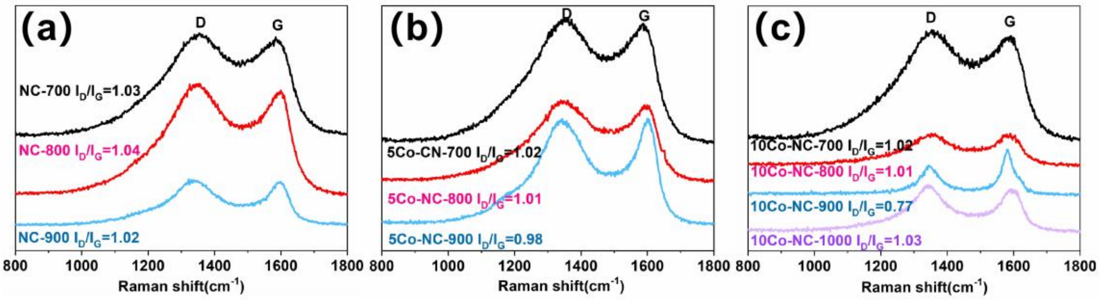

Figure S4. Raman spectra of Co-NC catalysts synthesized with various calcination temperatures and Co loadings.

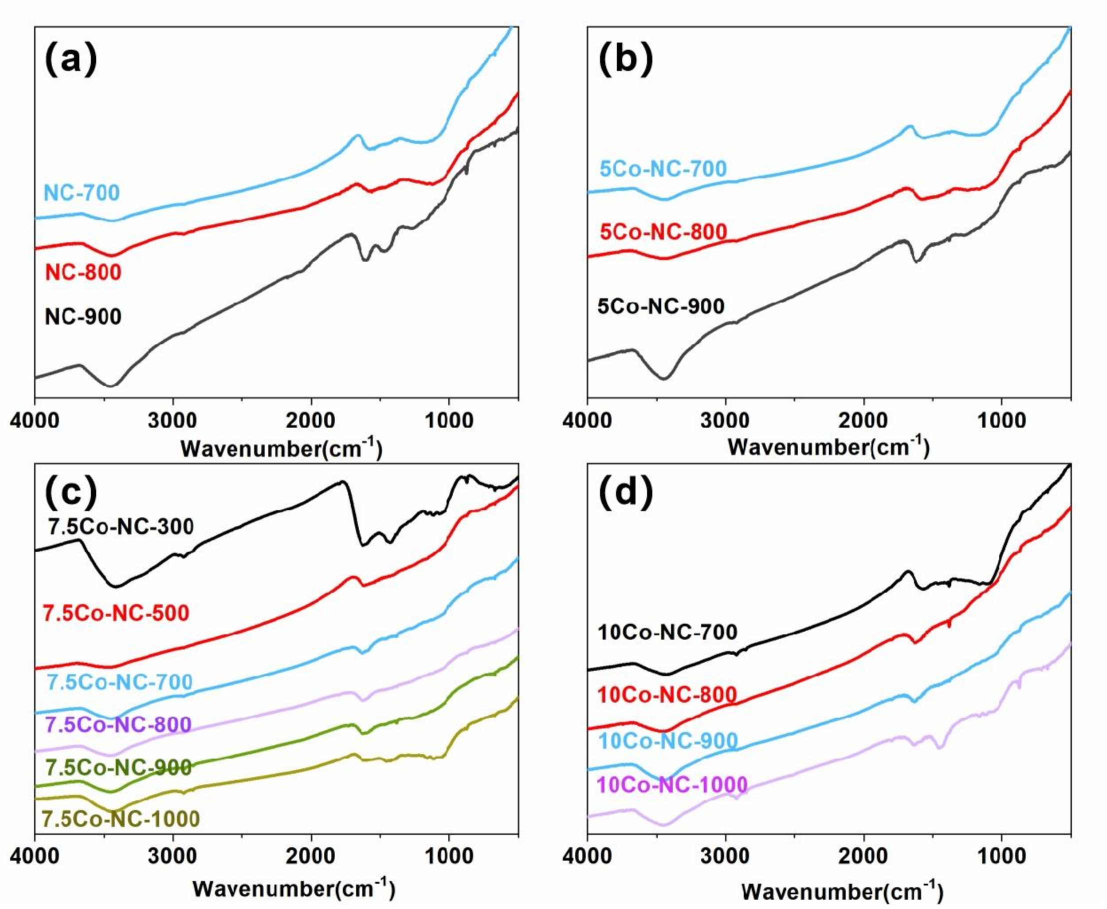

Figure S5. Infrared spectra of Co-NC catalysts synthesized with various calcination temperatures and Co loadings.

Table S1. The content of nitrogen in various catalysts.

No.	Sample	The content of nitrogen
1	5Co-NC-900	2.8%
2	7.5Co-NC-300	8.3%
3	7.5Co-NC-500	5.2%
4	7.5Co-NC-700	3.2%
5	7.5Co-NC-800	2.8%
6	7.5Co-NC-900	2.7%
7	7.5Co-NC-1000	2.1%
8	10Co-NC-900	2.4%
